# Snacks, beverages, and physical activity during volunteer-led out-of-school-time programs: a cross-sectional analysis

**DOI:** 10.1186/s12889-017-4040-2

**Published:** 2017-01-27

**Authors:** Christina D. Economos, Stephanie Anzman-Frasca, Alyssa H. Koomas, Grace Chan, Sara C. Folta, Julianne Heck, Molly Newman, Jennifer M. Sacheck

**Affiliations:** 1 ChildObesity180, Gerald J. and Dorothy R. Friedman School of Nutrition Science and Policy, Tufts University, 150 Harrison Avenue, Boston, 02111 MA USA; 2 Gerald J. and Dorothy R. Friedman School of Nutrition Science and Policy, Tufts University, 150 Harrison Avenue, Boston, MA 02111 USA; 3 Department of Pediatrics, Jacobs School of Medicine and Biomedical Sciences, University at Buffalo, G56 Farber Hall, South Campus, Buffalo, NY 14214 USA

**Keywords:** Out-of-school time, After-school programs, Enrichment, Youth sports, Children, Snacks, Beverages, Nutrition, Physical activity

## Abstract

**Background:**

Tens of millions of children regularly participate in out-of-school-time (OST) programs, providing an opportunity for child health promotion. Most research on OST has focused on structured, staff-led after-school programs, as opposed to volunteer-led programs such as enrichment programs and youth sports. The aim of this study was to describe snacks, beverages, and physical activity (PA) practices in volunteer-led OST programs across five organizations in three states.

**Methods:**

An online survey including the Out-of-School-Time Snacks, Beverages, and Physical Activity Questionnaire was distributed to 1,695 adult leaders of enrichment and youth sports programs serving 5–12 year-old children in Maine, Massachusetts, and New Hampshire, USA. The response rate was 57.8%, with 980 leaders participating and 698 (136 youth sports, 562 enrichment) remaining after data cleaning procedures. Frequencies were calculated to describe snack, beverage, and PA offerings during typical meetings and whether healthy snack, beverage, and PA criteria were met. Criteria were developed a priori with the intent to capture co-occurring practices that together indicate healthy snack (fruits and vegetables or no snack over salty/sweet snacks); beverage (water over sugar-sweetened beverages); and PA environments (regular opportunities for >15 or 45 min of PA in enrichment and sports programs, respectively).

**Results:**

About half of enrichment leaders reported that snacks and beverages were provided during typical meetings vs. one-fifth of sports leaders. In 28.4% of enrichment programs, PA was offered at every meeting vs. 98.5% of sports programs. Among enrichment programs, 50.4 and 25.8% met healthy snack and beverage criteria, respectively, and 29.4% met PA criteria, with 27.6% meeting criteria in two or more areas, and 5.0% in all three. Among sports programs, 72.8 and 78.7% met healthy snack and beverage criteria, respectively, and 71.3% met PA criteria. Eighty-two percent met criteria in two or more areas, and 46.3% met criteria in all three.

**Conclusions:**

Most programs did not meet criteria for healthier snacks and beverages and opportunities for PA during typical meetings, indicating room for improvement in encouraging widespread adoption of these practices. Efforts to improve the healthfulness of snacks and beverages and increase opportunities for PA during volunteer-led OST programs are warranted.

**Electronic supplementary material:**

The online version of this article (doi:10.1186/s12889-017-4040-2) contains supplementary material, which is available to authorized users.

## Background

Out-of-school-time (OST) programs offer an opportunity to promote healthy eating and physical activity (PA) to children. OST settings include structured, staff-led after-school programs, as well as volunteer-led enrichment and youth sports programs that take place before and after school, during school vacations, and on weekends [1–6]. Volunteer-led OST programs vary in mission, from scouting programs that aim to instill characteristics of leadership and civic duty, to youth sports programs that focus on physical health and teamwork among other skills. Tens of millions of children participate in volunteer-led OST programs, including nearly six million in 4-H, more than four million in scouting programs, and three million in US Youth Soccer [7–10]. These programs merit specific study because health promotion in volunteer-led OST programs may require different tactics than staff-led programs due to unique constraints, such as less control over policy implementation, less leader training, high turnover and frequent reliance on parent volunteers.

Overall, studies of nutrition and PA practices in OST programs have highlighted room for improvement, with many programs offering energy-dense, nutrient-poor snacks, and a small percentage of program time utilized for PA [4, 11–15]. Recent evaluations of interventions and policies aiming to improve these practices have shown promise in structured, staff-led after-school programs [4, 13, 16–21]. Fewer studies have examined snack, beverage, and PA offerings in volunteer-led OST programs. Research on youth sports’ role in obesity prevention found youth sport participants were more likely to consume fast food and drink sugar-sweetened beverages than non-participants and found no clear association between body weight and sport participation [22]. Insight into food and beverage offerings during youth sports may help to explain these results. Currently, studies measuring snack, beverage, and PA offerings in volunteer-led OST programs on a large scale (e.g., by assessing a sizeable sample of OST programs from multiple national organizations and states) are lacking. Understanding current practices in volunteer-led OST programs can inform ongoing efforts to promote healthy environments for children and may reveal opportunities for these settings to contribute to childhood obesity prevention efforts.

In 2011 the National AfterSchool Association adopted voluntary, evidence-based, Healthy Eating and Physical Activity (HEPA) Standards for OST settings [23]. These comprehensive standards are intended for structured after-school programs with paid staff and employee training programs; volunteer-led OST programs are more likely to have barriers to full adoption of these standards due to volunteer constraints (e.g., time, training) and a lack of logistical supports (e.g., reliance on parent-provided snacks, lack of kitchen facilities). Therefore, a need was identified for simple, easy-to-implement principles designed to support volunteer-led OST programs in creating healthier environments [24]. The Healthy Kids Out of School (HKOS) initiative facilitated development of these principles in 2011 with input from a group of nine national OST organizations (Boy Scouts of America, US Youth Soccer, Pop Warner, Boys and Girls Clubs of America, YMCA of the USA, Girl Scouts of the USA, National Council of Youth Sports, National Council of La Raza, and the National Urban League) [24]. These organizations were selected due to their large national reach and, ultimately, due to their commitment at the leadership level to disseminate healthy program guidelines. Following this effort, HKOS developed programs and training materials to support volunteer-led enrichment and youth sports OST programs in adopting three evidence-based principles for obesity prevention: *Drink Right:* Choose water over sugar-sweetened beverages; *Move More:* Boost movement and physical activity in all programs; and *Snack Smart:* Fuel up on fruits and vegetables [25].

The goal of the current study was to assess the extent to which these healthy practices of offering water as the primary beverage, fruits and vegetables as snacks, and frequent opportunities for PA were being met during both enrichment (Boy Scouts, 4-H) and youth sports (Youth Soccer, Pop Warner Football and Cheerleading, YMCA youth sports) programs before the implementation of any HKOS interventions promoting them. Understanding current practices in these large volunteer-led OST programs can elucidate opportunities to improve nutrition and PA practices for millions of children and provide data against which future intervention efforts can be evaluated.

## Methods

### Participants

An online survey was distributed to 1,695 OST leaders of enrichment or youth sports programs in Maine, Massachusetts, and New Hampshire, USA. Study staff collaborated with regional OST administrators to identify leaders, obtain contact information, and enlist support in survey distribution prior to the implementation of any HKOS interventions. Program leaders affiliated with one of five OST organizations (Boy Scouts of America, 4-H, US Youth Soccer, Pop Warner Football and Cheerleading, YMCA youth sports) within the indicated states who were ≥18 years old and who worked directly with at least some children between the ages of 5 – 12 were eligible to complete the survey. Nine hundred and eighty leaders participated (57.8% of survey recipients), with 698 remaining in the sample after data cleaning procedures described below (562 enrichment, 136 sports). Leaders were 94% non-Hispanic White in enrichment programs and 88% non-Hispanic White in sports programs, with a mean age of 48.0 years (SD = 9.5) and 42.1 years (SD = 6.3), respectively. Enrichment programs had more boys than girls participating as one of the two enrichment programs (Boy Scouts) included only boys. Sports programs consisted of both boys and girls (median = 60.0% boys; see Table 1). In both types of programs, children reached were predominantly non-Hispanic White. Additional child characteristics are described in Table 1. All human subjects procedures were performed in accordance with the Declaration of Helsinki and were approved by the Tufts University Institutional Review Board.

**Table 1:** OST program-level demographics

	Enrichment	Sports
Sample size (number of OST programs)	562	136
OST organizations represented	Boy Scouts (391), 4-H (171)	Soccer (125), YMCA (2), Pop Warner (9)
# of children per program, median (IQR)	14 (17)	12 (3)
Duration of typical program meeting	6.1% <1 h; 83.4% 1- < 2 h; 10.2% 2 h or more	8.3% <1 h; 86.5% 1- < 2 h; 5.3% 2 h or more
Sex of children		
% boys, median (IQR)	100 (50)	60 (100)
% girls, median (IQR)	0 (50)	40 (100)
Race of children^a^		
% White, median (IQR)	99.8 (10)	90 (20)
Age of children^b^		
% <5 years, median (IQR)	0 (0)	0 (0)
% 5–7 years, median (IQR)	0 (25)	0 (0)
% 8–12 years, median (IQR)	50 (65)	100 (10)
% 13 and older, median (IQR)	5.5 (60)	0 (0)

Demographic data were program leader-reported. Medians and interquartile ranges are reported where distributions were non-normal. For child characteristics shown in bold, leaders reported the percentage of the children in their program in each category. Medians depicted for those variables reflect the median percentage reported across programs. Numbers of leaders reporting on these variables were as follows: *n* = 530 enrichment, *n* = 133 sports. Missing data were due to a small number of respondents exiting the survey before its completion

^a^Number of programs reporting children of each race/ethnicity other than White: African-American (*n* = 180), Hispanic (*n* = 157), Asian (*n* = 162), Other (*n* = 103)

^b^Inclusion criteria for recruitment specified that programs should serve at least some children 5–12 years

### Procedures

**Fig. 1:**
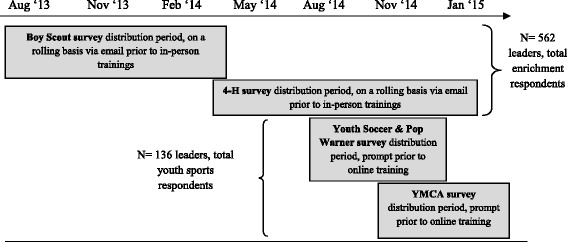
Survey distribution period and method for each OST organization spanning from August 2013 through January 2015 is shown

A 44-item survey was administered online using Qualtrics software (Qualtrics, Provo, UT). The survey was disseminated on a rolling basis to assess typical snack, beverage, and PA offerings. For enrichment programs (Boy Scouts, 4-H), a link to the online survey was distributed via email to leaders prior to an HKOS training beginning in August 2013 for Boy Scouts and March 2014 for 4-H. For youth sports (Youth Soccer, Pop Warner, YMCA youth sports), recruitment took place via a prompt prior to taking an online training for coaches immediately prior to their sport’s season: August – November 2014 for Youth Soccer and Pop Warner, and October 2014 – January 2015 for YMCA youth sports. Because distribution of the survey was linked to the enrichment program year (academic year) and youth sports season (2–4 month seasons), enrichment programs had a longer period of data collection compared to youth sports (Fig. 1).

The online survey consisted of the Out-of-School-Time Snacks, Beverages, and Physical Activity Questionnaire (OST-SBPA), as well as additional questions about program-level demographics, beverages brought to typical program meetings by individual children, and snacks/beverages available outside of typical program meetings. The OST-SBPA measures nutrition and PA offerings as reported by the program leader, focusing on program-provided snacks, beverages, and PA opportunities during typical OST program meetings [26]. Typical meetings were defined as regular meeting times for enrichment programs and as practices for youth sports, excluding games, awards ceremonies, camps, tournaments, and other meetings deemed “special events”. The OST-SBPA includes two categorical items about the provision of snacks and beverages respectively, with response options as follows: there are no snacks/beverages during typical meetings, or snacks/beverages are provided to the group and/or are brought by individual children. An additional response option for beverages is that water is available to children via water fountains. These items are followed by 13 items with continuous response scales, which assess the frequency that specific categories of snacks and beverages are served (among programs with program-provided snacks/beverages) and the frequency and duration of opportunities for PA. Specific snack and beverage categories are shown in Additional file 1: Appendix A, and all snack, beverage, and PA questions and response options reported herein are shown in Additional file 2: Appendix B. Support for the criterion validity of the OST-SBPA was previously demonstrated across a range of OST programs including volunteer-led enrichment and youth sports programs [26].

### Data analysis

#### Data cleaning

Leaders’ survey data were exported from Qualtrics to SAS (Cary, NC). Program-specific identifiers (e.g., state, town, club name) were concatenated to form ID variables at the program level. A phased de-duplication procedure was enacted in order to identify and remove duplicate observations at the program-level, so that no OST programs would be over-represented in the data (e.g., if two leaders from the same program responded about that program). This process began with an automated phase, using a SAS program to identify duplicates, followed by a manual check. In cases of clear duplicates, the observation that was recorded first was retained unless that observation was missing data on snacks, beverages, and PA, in which case this rule was overridden, and the next observation was retained. There were some cases of unclear duplicates (less than 1% of cases per OST organization); given the uncertainty as to whether these were true duplicate observations and the fact that they represented a small minority of observations, these cases were retained. After de-duplication, remaining data cleaning steps included examining distributions and outliers and recoding implausible values (e.g., a birth date entered as the implausible value of 1075 was recoded as the likely correct response: 1975). The final cleaned dataset was restricted to include only programs with at least one child in the age range of interest (5-12 years), given that this was an intended inclusion criterion, and was also restricted such that cases in which leaders did not respond to at least one survey item about snacks, beverages, and/or PA were excluded.

#### Frequencies on individual questions of interest

Frequencies were calculated by organization type (enrichment vs. youth sports), with variables of interest including program-provided snack, beverage, and PA variables from the OST-SBPA [26], as well as beverages provided by individual children and snacks and beverages served during special events.

#### Creating composite “success indicators”

Additionally, composite variables were created to provide a more comprehensive indication of healthy snack, beverage, and PA practices. These success indicators were developed a priori by the research team with the intent to capture co-occurring practices that together indicate healthy snack, beverage, and PA environments as defined by the evidence-based HKOS principles [26] (e.g., water being served in the absence of sugary beverages indicates a healthy beverage environment, while water served alongside sugary beverages does not). Success indicators were defined as follows, and frequencies were calculated to determine the percentage of programs that were successful in each area:

1. Snack success was defined as follows:
  No snacks at meetings, or
  If the program provided snacks: (1) Fresh or processed fruits/vegetables were served at most or all meetings, and salty and sweet snacks were served at only some or no meetings, or (2) Fresh or processed fruits/vegetables were served at some meetings and salty and sweet snacks were served at no meetings.
2. Beverage success was defined as follows:
  If the program provided beverages: Water was provided at most or all meetings, and sugar-sweetened beverages were provided at no meetings.
  If individual children brought beverages: Water was brought to most or all meetings and sugar-sweetened beverages were brought to only some or no meetings.
3. PA success was defined as follows:
  Enrichment programs offered opportunities for PA at most or all meetings, and when PA was offered, it was for >15 minutes per meeting.
  Sports programs offered opportunities for PA at every meeting, and when PA was offered, it was for >45 minutes per meeting.

## Results

Among the 562 enrichment program leaders, 55.0% reported that the program provided a snack to the group, 8.6% reported that children brought their own snacks, and 39.2% reported that snacks were not eaten during typical meetings. The former two options were not mutually exclusive. For sports, among the 126 leaders, 21.3% reported that the program provided snacks to the group during typical meetings (defined here as practices), 21.3% reported that individual children brought snacks, and 61.0% reported that snacks were not eaten during typical meetings. Given that most sports programs indicated that snacks were not provided for the group during typical meetings and the subsequent skipping of survey items about specific snack categories, there was a substantially smaller sample for these items (see Table 2 for specific frequencies and sample sizes).

**Table 2:** Percentages of programs with specific snack and beverage types served/brought during typical meetings

	Enrichment programs^a^			Sports programs^a^		
	None of the times we meet	Some of the times we meet	Most/every time we meet	None of the times we meet	Some of the times we meet	Most/every time we meet
Snacks provided for group						
	*n* = 299-303			*n* = 29		
Fresh FV	16.8	51.5	29.0	10.3	31.0	48.3
Processed FV	43.1	46.7	7.0	72.4	6.9	3.5
Salty snacks	15.2	52.7	30.8	55.2	17.2	6.9
Sweet snacks	35.1	51.7	10.6	65.5	10.3	3.5
Protein	56.9	31.1	7.4	69.0	6.9	3.5
Beverages provided for group						
	*n* = 275-278			*n* = 26		
Water	3.6	18.4	77.3	0.0	7.7	92.3
Milk	69.1	19.6	8.0	84.6	3.9	0.0
Juice	20.9	44.6	32.0	61.5	15.4	3.9
SSB	43.7	35.4	18.8	46.2	23.1	11.5
Beverages brought by children^b^						
	*n* = 75			*n* = 127		
Water	1.3	28.0	69.3	0.0	0.8	99.2
Milk	77.3	14.7	1.3	95.3	0.8	0.8
Juice	34.7	48.0	10.7	73.2	14.2	1.6
SSB	44.0	38.7	10.7	40.2	44.9	8.7

^a^Sample sizes reported in the table vary with missing data mostly due to some of the 562 enrichment programs and 136 sports programs not serving snacks or beverages as described in the Methods section, and a few additional missing cases due to non-response. A small number of leaders responded “Don’t Know” to these items as opposed to selecting one of the frequencies indicated above; therefore the percentages above may not total 100%

^b^It is uncommon for sports programs to provide snacks or beverages to the group during typical meetings (i.e. practices) although children do drink beverages during these meetings. For this reason, we also examined beverages brought by children within the beverage categories common among sports programs (water, SSB)

Note: Detailed information on the creation and contents of the snack/beverage categories is available here. [26] *FV*: fruits/vegetables. *SSB*: sugar-sweetened beverages

For beverages, 49.4% of enrichment program leaders reported that program-provided beverages were served to the group, 14.2% reported that individual children brought their own beverages, 22.6% reported that children drank water from water fountains, and 25.8% reported that children did not drink beverages during typical meetings. Among sports programs, 19.1% of leaders reported that program-provided beverages were served to the group, 93.4% reported that individual children brought their own beverages, 1.5% of leaders reported that children drank from water fountains, and 0% reported that children did not drink beverages during typical meetings (practices). Given that most sports programs indicated that beverages were not provided for the group during typical meetings and the subsequent skipping of survey items about specific beverage categories, there was a substantially smaller sample for these items (see Table 2 for specific frequencies and sample sizes). We also examined the extent to which specific beverages were brought to typical meetings by individual children after noting that individually-provided beverages were common in sports (Table 2). Of those leaders who reported that children provide their own beverages, over half reported that sugar-sweetened beverages were provided at least some of the times they meet.

**Fig. 2:**
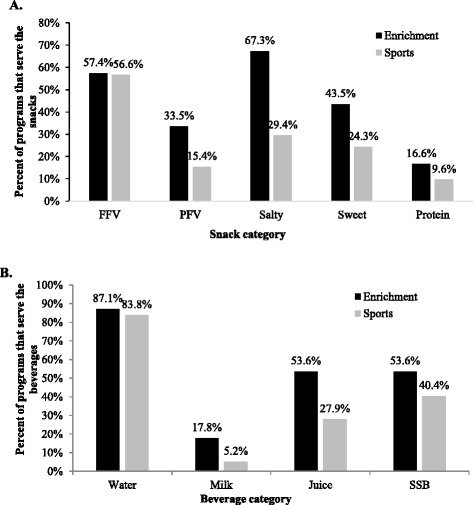
Snacks and beverages served at special events. The percentages of enrichment and sports programs serving snacks and beverages in each category of interest at special events, such as games, tournaments, camps, and celebrations, are shown. A dichotomous response was recorded for each snack and beverage type, indicating whether or not it was ever served at a program’s special events; thus, snack/beverage types are not mutually exclusive. The number of leaders indicating that they served each snack and beverage type was divided by the total number of programs with data on snacks (*n* = 547 enrichment, 136 sports) and beverages (*n* = 556 enrichment, 136 sports) at special events, respectively. FFV = fresh fruits and vegetables, PFV = processed fruits and vegetables, SSB = sugar-sweetened beverages

When asked whether each snack/beverage category was ever available at special events, the most common categories reported by enrichment leaders were salty snacks and water. Among sports leaders, the most common categories reported were fresh fruits and vegetables and water, although the following were also reported by ~25% or more of sports leaders: salty snacks, sweet snacks, juice, and sugar-sweetened beverages (see Fig. 2).

Leader reports of PA opportunities provided during typical program meetings appear in Table 3. Among enrichment programs, it was most common for PA to be offered at most meetings (36% of programs). When PA was offered, it was most common for it to be 1–15 min in duration and for all children to participate. Among sports programs, nearly all leaders reported that PA was offered at every meeting, with the most commonly reported duration of PA opportunities being 46–60 min, and with all children participating (see Table 3).

**Table 3:** Physical activity during typical meetings of enrichment and sports programs

	Enrichment	Sports
	% of programs	% of programs
Frequency of PA across typical meetings		
None of the times we meet	7.0	0.0
Some of the times we meet	28.0	0.0
Most of the times we meet	36.2	1.5
Every time we meet	28.4	98.5
Duration of PA opportunities when offered^a^		
1–15 min	58.6	2.2
16–30 min	30.3	6.6
31–45 min	3.8	19.1
46–60 min	1.4	41.9
60+ min	1.0	29.4
Proportion of children participating when PA is offered (reach)		
Some children	2.4	0.7
Most children	30.7	8.1
All children	65.3	90.4

Sample sizes for the above are as follows: enrichment: *n* = 495–542, sports: *n* = 136. Missing enrichment data are mostly due to skip patterns with a small number of cases missing due to non-response

A small number of leaders responded “Don’t Know” to these items; therefore the percentages above may not total exactly 100%

^a^Reported program meeting durations appear in Table 1

*PA*: physical activity

**Fig. 3:**
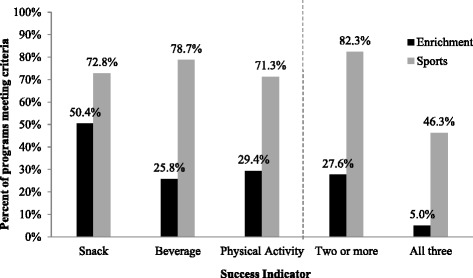
Percent of programs meeting pre-defined success indicators for healthy snack, beverage, and physical activity practices. This figure depicts the percentage of all enrichment (*n* = 562) and sports (*n* = 136) programs meeting specified evidence-based criteria for healthy snack, beverage, and physical activity practices, as well as the percentage meeting two of the three and all three sets of criteria. Snack success was defined as either: (1) providing fresh or processed fruits/vegetables to the group at all or most meetings and salty and sweet snacks at no or only some meetings, (2) providing fresh/processed fruits/vegetables at some meetings and salty/sweet snacks at no meetings, or (3) not serving a snack. Beverage success was defined as: (1) providing water to the group at all or most meetings and never providing sugar-sweetened beverages, or (2) having water brought by individual children at all or most meetings and sugar-sweetened beverages brought at no or only some meetings; if programs had both group-provided and individually-provided beverages, both of these conditions needed to be met to receive credit for beverage success. Physical activity success was defined as: offering opportunities for physical activity at most or every meeting time, with physical activity opportunities lasting more than 15 min when offered in enrichment programs and more than 45 min in sports programs

When examining the percentage of programs meeting pre-defined criteria for healthy snack, beverage, and PA practices during typical meetings (the success indicators), 27.6% of enrichment programs met criteria in at least two of these three areas, and only 5% met all three. Among sports programs, 82.3% met criteria in at least two of these areas, and 46.3% met criteria in all three. The percentage of programs meeting each specific criterion is shown in Fig. 3.

## Discussion

This study demonstrates that volunteer-led OST programs are important settings for child health promotion. Findings showed that less than half of sports programs and only 5% of enrichment programs met criteria for healthy snack, beverage, and PA offerings during their typical program meetings. Similar results have been documented in structured, staff-led after-school programs, with many programs failing to provide fruits, vegetables, and water on a regular basis and demonstrating challenges meeting PA guidelines [16, 27]. Studies of health policy implementation in staff-led OST programs have revealed improved nutrition and PA outcomes, suggesting volunteer-led OST programs could also benefit from such interventions [28, 29]. Yet to date, interventions to promote nutrition and PA guidelines to volunteer-led OST programs have not been undertaken. Together, the current findings and the extant literature suggest that there is significant room for improvement in encouraging more widespread and consistent adoption of healthy eating and PA practices in volunteer-led OST programs though the specific approach to successfully encourage these practices may differ by program type.

Some differences between enrichment and sports programs were observed in this study. Overall, each of the three healthy habits of interest (provision of fruits and vegetables or no snacks over salty or sweet snacks, water over sugar-sweetened beverages, and regular opportunities for PA) was reported by less than half of enrichment leaders, with only 5% of leaders reporting that all three of these were in place during typical meeting times. More than 70% of sports leaders reported success with each of these three habits, demonstrating a higher prevalence of each individually, although less than half of leaders reported success with all three.

### Enrichment programs

Among enrichment programs, the current findings indicate that a variety of snacks are commonly provided by programs during typical meetings, including both salty/sweet and fruit and vegetable snacks. While water was regularly provided by the majority of enrichment programs, sugar-sweetened beverages and juice were common with over one-half of leaders reporting on program-provided beverages indicating that sugar-sweetened beverages are served at least some of the time. A reduction in nutrient-poor, higher-calorie snacks and beverages provided in these settings could significantly impact children’s energy balance given that enrichment programs tend to meet over the course of a full academic year, with many children participating over multiple program years. Healthy habits learned through repetition in these settings may also extend to other settings [30, 31]. The finding that some troops and clubs have already found it feasible to serve fruits and vegetables suggests an opportunity for future research to examine key factors that have allowed for healthy programs and to share successful strategies with other programs not yet achieving these standards.

Similarly, findings pertaining to PA suggest that incorporation of PA is feasible within enrichment program meetings and indicate opportunities to promote more widespread adoption of this practice through activities like games and “active breaks”. While some enrichment leaders reported that PA is not a part of typical meetings, the majority reported that PA is offered regularly, with the most commonly-reported amount of PA time offered during typical meetings being 1–15 min. These findings highlight the opportunity for more enrichment programs to include PA and for most to increase total minutes of PA.

Health promotion efforts during enrichment programs may influence children’s long-term health behaviors due to the lasting impact of the values instilled during these types of programs. For instance, it has been found that both adult Girl Scout and Boy Scout alumni display positive life outcomes historically cultivated by these programs, including a commitment to community service and civic engagement [32, 33]. If the leaders who teach and role model these values begin to champion healthy eating and PA, these messages could translate into sustained healthy habits later in life.

### Sports programs

As defined in this study, “typical meetings” capture the majority of enrichment program gatherings while sports programs’ “typical meetings” were defined as practices, which are one of two types of regular sports program gatherings, the other being games. Our results showed that many sports programs do not typically serve a snack during practices, and that water provided by individual children was the most commonly-offered beverage during these meetings. Physical activity in sports programs was high with nearly every team reporting PA at every meeting, and the majority offering 46 min or more. While these findings fit with pre-defined “success” criteria used in this study, results also suggest that it is important to target and monitor the food environment at games and other special events, where snacks and beverages are more likely to be available to youth sports participants. Sixty-eight percent of sports leaders reported that at least one type of snack was served at special events, and while fruits and vegetables were the most common offering, substantial percentages of leaders reported that salty and sweet snacks were available. Similarly, water was the most common beverage at special events, but substantial percentages of sports program leaders reported the availability of juice and sugar-sweetened beverages. Games take place more frequently (typically at least weekly) compared to enrichment programs’ special events, providing further rationale for incorporating them into youth-sports-focused health promotion efforts. Emerging research suggests that healthier options sold in concession stands during youth sports resulted in reduced sugar-sweetened beverage intake [34] and may not impact sales or customer satisfaction [35]. Together, findings suggest that a focus on program-provided snacks and beverages is appropriate when studying enrichment programs, while a more comprehensive focus, including beverages brought to practices by individual children and snacks and beverages provided during games, is warranted among sports programs.

Limitations of the current study include the possibility of social desirability biasing survey responses, as leaders may have over-reported practices that they perceived as desirable, such as healthier snacks and regular PA opportunities. The OST-SBPA instrument was validated against direct observation, attenuating this concern [26]. Additional research is needed to shed light on the measurement characteristics of the items used to assess individually-provided beverages and special events in this study, especially given their ubiquity among sports programs as discussed above. Other study limitations include the use of a convenience sample and response rates that differed by organization: in particular, there was a greater number of responses from one of the sports organizations versus the others. The overrepresentation of a particular sport could limit the generalizability of results, as food and PA environments may vary from sport to sport. Finally, the present sample is primarily non-Hispanic White. At the national level, these OST programs serve a more diverse population of children. For example, 4-H reaches almost two million Latino, Black, and Asian youth [7]. Subsequent research with volunteer-led OST programs in other geographic areas and with specific outreach programs [36, 37﻿] can further elucidate the generalizability of the current results to different populations.

Key strengths of this study include the use of validated survey items to assess snack, beverage, and PA practices impacting a large number of children attending volunteer-led OST programs from five national organizations. The ~700 OST leaders surveyed represent ~10,000 children participating in their programs (see Table 1). Compared to the literature on structured, staff-led after-school programs, there is less information available about nutrition and PA within volunteer-led OST programs. These programs are relevant from a public health standpoint given the large population of children regularly participating in these programs, many for multiple years, and the potential for repeated exposure to healthy habits during meetings that may generalize to other settings, effects that can be estimated using approaches like agent-based modeling [38﻿].

The current results provide necessary descriptive information about food and PA environments in volunteer-led OST settings, contributing to the overarching knowledge base about OST, and also highlight implications of the unique attributes of different OST settings. Specifically, findings suggest that intervention efforts within enrichment programs may be most successful if targeting leaders who are providing snacks and beverages and planning PA curricula for typical meetings. Intervention efforts targeting sports programs should consider messages at the child and parent level given the prevalence of individually-provided beverages and should consider widening their focus to games. In sum, these results provide insights into the nutrition and PA environments of volunteer-led OST programs that serve millions of children and highlight considerations for future research, including the implementation and evaluation of health promotion efforts within these programs.

## Conclusions

The current study highlights room for improvement among both enrichment and sports programs, indicating that interventions aimed at improving the healthfulness of snacks and beverages, as well as increasing opportunities for and duration of PA during volunteer-led OST programs, are warranted. Findings also revealed variability in snack, beverage, and PA offerings between different OST organization types, which can inform the targeting of such intervention efforts. Evidence that some programs are already serving healthy snacks and beverages and offering ample opportunities for PA is promising for intervention efforts attempting to make these behaviors more widespread and normative across OST programs. Given that OST programs have the potential to reach millions of children nationwide, an understanding of current food, beverage, and PA practices across OST program types is a critical step toward increasing the effectiveness of OST programs as venues to reinforce healthy habits and help prevent childhood obesity. Leveraging OST programs’ reach and influence to consistently address physical health can transform these environments into settings promoting whole-child health and well-being, building upon OST organizations’ traditional focus areas, such as the promotion of leadership skills, teamwork, and civic engagement.

## Additional files

Additional file 1: Description of key snack, beverage, and PA items. (DOCX 18 kb)
Additional file 2: OST-SBPA items and additional questions. (DOCX 19 kb)

## Abbreviations

HEPA: Healthy Eating and Physical Activity Standards for out-of-school-time settings
HKOS: The Healthy Kids Out of School initiative
OST: Out-of-school-time
OST-SBPA: The out-of-school-time snacks, beverages, and physical activity questionnaire
PA: Physical activity
SD: Standard deviation
