# Supplementary material for: Snacks, beverages, and physical activity during volunteer-led out-of-school-time programs: a cross-sectional analysis
Source: BMC Public Health. 2017 Jan 27;17:125. doi: 10.1186/s12889-017-4040-2 (PMC5270327; doi:10.1186/s12889-017-4040-2)
Supplement: Additional file 1: — Description of key snack, beverage, and PA items. (DOCX 18 kb) [file 12889_2017_4040_MOESM1_ESM.docx]

**Appendix A. Description of key Snack, Beverage, and PA items**

| Categories | Described in questionnaire as: |
| --- | --- |
| Snack categories:  Fresh F&V | Snack category 1: Fresh F&V like apples, oranges, celery, and carrots |
| Processed F&V | Snack category 2: F&V with dips or sauces, canned fruit or vegetables, fruit cups or applesauce, and dried fruit like raisins |
| Salty snacks | Snack category 3: Snacks like Doritos or Sun Chips, pretzels, popcorn, crackers like Cheez-Its, Goldfish or Wheat Thins, or tortilla chips |
|  |  |
| Sweet snacks | Snack category 4: Snacks like ice cream, pudding, cookies, donuts, candy, granola or cereal bars, graham crackers, and breakfast cereals |
| Nut/bean/dairy snacks | Snack category 5: Snacks like nuts and nut butters, yogurt, cheese, cottage cheese, beans, and hummus |
| Beverage categories: |  |
| Water  Milk  Juice  Sweetened beverages  Aspects of physical activity  Frequency  Percent time  Duration  Reach | Beverage category 1: Water  Beverage category 2: Milk  Beverage category 3: 100% juice like apple juice or orange juice  Beverage category 4: Sweetened beverages like regular or diet soda, flavored iced teas, sports drinks, and juice drinks like Capri Sun or Kool Aid  How often opportunities for physical activity are offered  Proportion of program time children are active  How many minutes of physical activity during program session  How many children participate when opportunities for physical activity are offered |
|  |  |

Note: Respondents were asked about the frequency that the different categories of snacks and beverages were served across typical OST program sessions, as well as information about 4 different aspects of OST physical activity.
