# Supplementary material for: Snacks, beverages, and physical activity during volunteer-led out-of-school-time programs: a cross-sectional analysis
Source: BMC Public Health. 2017 Jan 27;17:125. doi: 10.1186/s12889-017-4040-2 (PMC5270327; doi:10.1186/s12889-017-4040-2)
Supplement: Additional file 2: — OST-SBPA items and additional questions. (DOCX 19 kb) [file 12889_2017_4040_MOESM2_ESM.docx]

**Appendix B. OST-SBPA Items and Additional Questions**

| **Question** | **Response options** |
| --- | --- |
| Who provides the snacks that children eat at typical program sessions? Select all that apply. | A. Staff, volunteers, parents, and/or the program provides snack(s) for the group  B. Children bring their own individual snacks  C. Children do not eat snacks at typical program sessions  D. Don't know |
| Thinking about typical program sessions, how often are the following snacks provided for the group? This may include snacks provided by staff, volunteers, and/or parents. *(Respondents complete this question for each snack category; Appendix A)*. | A. Every time we meet  B. Most of the times we meet  C. Only some of the times we meet  D. None of the times we meet  E. Don't know |
| Thinking about typical program sessions, how many children provide their own individual snacks? This may include snacks brought from home or purchased by the child (e.g., from a vending machine, concession stand, or convenience store). ^1^ | A. All children  B. Most children  C. Only some children  D. None of the children  E. Don’t know |
| When children provide their own individual snacks at typical program sessions, how often do they bring the following snacks? This may include snacks brought from home or purchased by the child (e.g. from a vending machine, concession stand, or convenience store). *(Respondents complete this question for each snack category; Appendix A)*. ^1^ | A. Every time we meet  B. Most of the times we meet  C. Only some of the times we meet  D. None of the times we meet  E. Don't know |
| Which of the following snacks are typically served at special events (sports games or tournaments, field trips, camps, award ceremonies, etc.) for your program? Select all that apply.^1^ | A. Snack category 1  B. Snack category 2  C. Snack category 3  D. Snack category 4  E. Snack category 5  F. Don’t know |
| Who provides the beverages that children drink at typical program sessions? This may include tap or bottled water, juice, milk, or other beverages. Select all that apply. | A. Staff, volunteers, parents, and/or the program provides beverage(s) for the group  B. Children bring their own individual beverages  C. Children drink from a water fountain, water cooler or bubbler where the program meets  D. Children do not drink beverages at typical program sessions  E. Don't know |
| Thinking about typical program sessions, how often are the following beverages provided for the group? This may include beverages provided by staff, volunteers, and/or parents. *(Respondents complete this question for each beverage category; Appendix A).* | A. Every time we meet  B. Most of the times we meet  C. Only some of the times we meet  D. None of the times we meet  E. Don't know |
| Thinking about typical program meetings, how many children provide their own individual beverages? This may include beverages brought from home or purchased by the child (e.g., from a vending machine, concession stand, or convenience store). ^1^ | A. All children  B. Most children  C. Only some children  D. None of the children  E. Don’t know |
| When children provide their own individual beverages at typical program meetings, how often do they bring the following beverages? This may include beverages brought from home or purchased by the child (e.g. from a vending machine, concession stand, or convenience store). *(Respondents complete this question for each beverage category; Appendix A)* ^1^ | A. Every time we meet  B. Most of the times we meet  C. Only some of the times we meet  D. None of the times we meet  E. Don't know |
| Which of the following beverages are typically served at special events (sports games or tournaments, field trips, camps, award ceremonies, etc.) for your program? Select all that apply. ^1^ | A. Beverage category 1  B. Beverage category 2  C. Beverage category 3  D. Beverage category 4  E. Don’t know |
| How often are opportunities for physical activity provided during typical program sessions? | A. Every time we meet  B. Most of the times we meet  C. Only some of the times we meet  D. None of the times we meet  E. Don't know |
| On average, what proportion of time are children physically active during typical program sessions? | A. They are active or moving just some or only a little of the time  B. They are active or moving most of the time  C. They are active or moving all or nearly all of the time  D. Don't know |
| On average, how many minutes of physical activity do children typically get during typical program sessions? | A. None  B. 1-15 minutes  C. 16-30 minutes  D. 31-45 minutes  E. 46-60 minutes  F. More than 60 minutes  G. Don't know |
| When opportunities for physical activity are provided during typical program sessions, how many children participate? | A. All children  B. Most children  C. Only some children  D. No children  E. Don't know |

^1^Additional snack and beverage questions that were administered along with the validated OST-SBPA Questionnaire

Note: Respondents were instructed to focus on typical program sessions, which were defined as follows. **For sports programs**: Typical program sessions include **practices only**. Please do NOT include games, tournaments, camps or other special events. **For all other programs**: Typical program sessions include **regularly scheduled program activities or meetings.** Please do NOT include field trips, camps, award ceremonies or other special events.
